# Variation in Protein and Calorie Consumption Following Protein Malnutrition in *Rattus norvegicus*

**DOI:** 10.3390/ani3010033

**Published:** 2013-01-24

**Authors:** Donna C. Jones, Rebecca Z. German

**Affiliations:** 1 Division of Plastic Surgery, ML 1020, Cincinnati Children’s Hospital Medical Center, Cincinnati, OH 45229, USA; 2 Department of Physical Medicine and Rehabilitation, Johns Hopkins University, 98 N. Broadway, Baltimore, MD 21231, USA; E-Mail: RZGerman@jhu.edu

**Keywords:** targeted growth, rodent ontogeny, catch-up growth

## Abstract

**Simple Summary:**

Catch-up growth following malnutrition is likely influenced by available protein and calories. We measured calorie and protein consumption following the removal of protein malnutrition after 40, 60 and 90 days, in laboratory rats. Following the transition in diet, animals self-selected fewer calories, implying elevated protein is sufficient to fuel catch-up growth, eventually resulting in body weights and bone lengths greater or equal to those of control animals. Rats rehabilitated at younger ages, had more drastic alterations in consumption. Variable responses in different ages and sex highlight the plasticity of growth and how nutrition affects body form. This work furthers our understanding of how humans and livestock can recover from protein-restriction malnutrition, which seems to employ different biological responses.

**Abstract:**

Catch-up growth rates, following protein malnutrition, vary with timing and duration of insult, despite unlimited access to calories. Understanding changing patterns of post-insult consumption, relative rehabilitation timing, can provide insight into the mechanisms driving those differences. We hypothesize that higher catch-up growth rates will be correlated with increased protein consumption, while calorie consumption could remain stable. As catch-up growth rates decrease with age/malnutrition duration, we predict a dose effect in protein consumption with rehabilitation timing. We measured total and protein consumption, body mass, and long bone length, following an increase of dietary protein at 40, 60 and 90 days, with two control groups (chronic reduced protein or standard protein) for 150+ days. Immediately following rehabilitation, rats’ food consumption decreased significantly, implying that elevated protein intake is sufficient to fuel catch-up growth rates that eventually result in body weights and long bone lengths greater or equal to final measures of chronically fed standard (CT) animals. The duration of protein restriction affected consumption: rats rehabilitated at younger ages had more drastic alterations in consumption of both calories and protein. While rehabilitated animals did compensate with greater protein consumption, variable responses in different ages and sex highlight the plasticity of growth and how nutrition affects body form.

## 1. Introduction

Catch-up growth is generated with realimentation of normal protein levels, regardless of sex and duration of malnutrition, although with variable results in bone growth and body mass [1]. Additionally, timing and duration of protein malnutrition leads to variations in phenotype, both in terms of final size, but also patterns of growth for different anatomical structures (e.g., eye, brain), even when tested on a consistent genetic background and with unlimited access to calories [1,2,3,4]. However, the mechanisms by which this occurs are not entirely understood. One facet of the physiologic mechanics is the *ad lib* consumption of both calories and protein as animals transition through these nutritional alterations. Our research documents how variation in the consumption of calories and protein led to differences in catch-up growth in body mass and long bones as a function of duration/timing. The data presented here are valuable because they are exactly the data that generated the growth and size patterns described earlier [1]. 

Based upon the catch-up growth rates of body mass and long bone length following the release of protein malnourishment in laboratory rats, we make several predictions regarding consumption of both calories and protein based upon the animals’ sex and timing/duration of malnutrition. First, because rehabilitation animals grow faster directly after the switch in protein relative to controls (*i.e.*, they experience catch-up growth), we predict that consumption through the dietary change will not alter and that the elevated growth rates will be supported by the increase in dietary protein, alone. Alternatively, it is possible that calorie intake increases with the rehabilitation diet to account for greater energy demands associated with increased growth rates. As there is a dose response in growth rates among treatment groups (*i.e.*, less time on a pathological diet is correlated with higher post-rehabilitation growth rates [1], we also predict that a similar dose effect will be associated with protein consumption: those animals being rehabilitated later in life will have a lower increase in protein consumption with the diet change. It is also possible that consumption of calories pre- and post-rehabilitation will be equal, elevating only the protein content of the rats’ diet, but that the alteration in macronutrient availability will be sufficient to sustain post-rehabilitation growth rates. Finally, we hypothesize that male and female animals will have different consumption rates, to accommodate their differential responses to relief from malnutrition.

## 2. Materials and Methods

### 2.1. Animal Husbandry

Husbandry for these animals has been described elsewhere [1] and all animal protocols were approved by the University of Cincinnati IACUC (#01-02-26-01). Briefly, 80 *Rattus norvegicus*, produced by dams fed standard laboratory chow (40 male, 40 female), were weaned to one of two isocaloric diets, a standard (SP) or reduced protein (RP) (Table 1). Animals were kept on a 12:12 light:dark cycle and housed in standard shoe box cages. Three experimental groups, labeled LP40, LP60, and LP90, were weaned to the reduced protein diet and switched to the standard protein diet at 40, 60, and 90 days of age, respectively. Once an animal was switched to the standard protein diet, the animal received that diet for the remainder of the experiment. Two other experimental groups were chronically fed standard (CT treatment) or reduced protein (LP treatment) diets for the duration of data collection (150 days). Each of the ten experimental groups had eight animals (two sexes × five diet treatments). Water was provided *ad libitum*, but food was rationed to monitor consumption levels. Animals were weighed daily (digital scale precise to 0.5 g) and radiographed every 2 to 3 days to monitor growth in body mass and long bone length, respectively [1].

**Table 1:** Contents by weight (g/kg) of reduced protein (SP) and standard protein (SP) diets. Table adopted from [1]. Diets were isocaloric, each providing 3.4 kCal/gram.

Ingredient	Reduced Protein	Standard Protein
Casein	46	276
Cornstarch	500.9	239.9
DYETROSE^® a^	167	110
Sucrose	100	100
Cellulose	50	50
Soybean oil	70	70
*t*-Butylhydroquinone	0.014	0.014
Salt mix #213266	35	35
Calcium phosphate dibasic	11.66	4.08
Calcium carbonate	3.91	9.49
Vitamin mix #310025	10	10
L-Cystine	0.7	4.1
Choline bitartrate	2.5	2.5
Blue dye	–	0.05

^ a ^DYETROSE^®^ (Dyets, Bethlehem, PA, USA) is selectively depolymerized food-grade cornstarch that can be substituted for corn-starch without any detectable dietary effects.

Each rat was provided with a measured quantity of food (either SP or RP), scaled to account for its sex and body mass, based on previous measurements of *ad libitum* consumption (Table 2) [2]. Each day, the remaining food was collected, weighed, and subtracted from the amount provided the previous day, providing a measure of the amount of food consumed (grams). The amount of protein consumed was calculated, as each diet had a known weight percentage of protein (RP = 4.6%, SP = 27.6%), as were the number of calories (3.4 kCal/gram for both diets).

**Table 2:** Daily food provision (g, ±1) as determined by animal body mass (g).

Males		Females	
body mass	food	body mass	food
≤59	9	≤59	9
60–69	10	60–69	11
70–99	17	70–79	14
100–109	18	80–89	16
110–139	21	90–109	17
140–189	22	110–119	18
190–239	24	120–129	20
240–289	27	130–149	21
≥290	32	150–189	23
		190–259	25
		≥260	27

During the experiment, some animals consumed all available food within 24 hours, leaving open the possibility that they may have consumed more if given the option. The number of days each animal consumed all food was counted and this was found to be limited: 33 animals had zero days, while only three animals exceeded ten days prior to reaching 135 days of age. Among males, this occurrence was randomly dispersed among treatment groups (*p* = 0.153; Kruskal-Wallis), but CT females did have fewer days of full consumption relative to the LP60 (*p* = 0.048) and LP90 animals (*p* = 0.043; Dwass-Steel-Chritchlow-Fligner test for pairwise comparisons following Kruskal-Wallis test result of *p* = 0.017). The majority of these instances (86.4%) were isolated to animals less than 40 days of age, when no group had yet been rehabilitated to full protein. During this time (22 to 39 days of age), there were no obvious differences in the growth of the LP60 and LP90 females relative to the LP and LP40 females [1], nor were there statistical differences in the amount of food consumed among any females eating the reduced protein diet. Due to the limited occurrence of this effect, we note the possibility of effect and consider any influence from this to be stochastic variation. It is also well established that *ad libitum* feeding usually results in over-fed rats [5], so it is likely that no rat was truly calorie restricted on a day when all of their food was consumed.

### 2.2. Data Analyses

All analyses utilized daily protein and kCal consumption standardized by body weight for each animal. Testing for a change in consumption across the rehabilitation time is challenging because consumption amounts of animals alter as a function of age, body size, and iterative daily influences unique to each animal [6]. To mediate for those daily fluctuations, we calculated a daily mean for each treatment group, from 22 to 150 days of age. To determine if treatment groups consumed different amounts of calories or protein during the duration of the experiment, data were analyzed with a mixed linear regression model, with age as a covariate and treatment as a fixed factor. Sexes were analyzed separately. If statistical differences were found, *post hoc* tests were conducted to determine the effect of treatment, utilizing a Bonferroni correction. Statistical significance was accepted at *p* ≤ 0.01.

Modeling consumption data per individual was complicated. While the LP and CT consumptions followed Gompertz curves with small amounts of variation [1,2,3,4], the rehabilitation groups had distinctive consumption curves that were difficult to model in a way similar to the CT and LP groups due to the extreme manipulation of protein content in the offered diets (from 4% protein to 27.6%). Therefore, we examined the data in two different ways. 

First, we tested average daily consumption (kCal or protein by body weight) within a treatment group over time, up to age 150 days. We analyzed these data, separated by sex, in a linear model, for 128 days, *i.e.*, starting at age 22 days, for 5 groups, with a sample of 640 daily consumption averages. The response variable in this model was average consumption per day. We included day as a random covariate, and tested for differences among our treatment groups. Thus significant differences reflect total consumption of either protein or total calories in two independent analyses. Additionally, males and females were analyzed separately or included as an additional fixed factor. 

**Figure 1:**
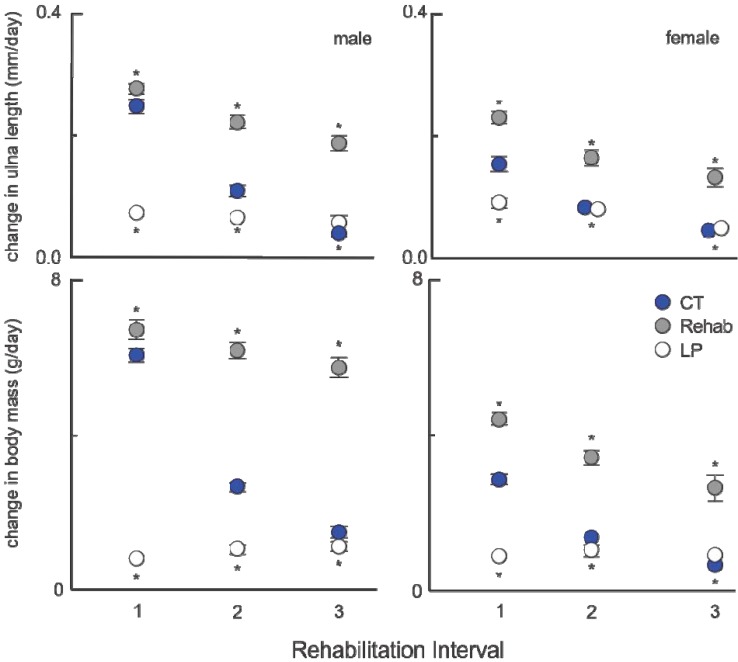
Post-rehabilitation growth (±standard deviation, n = 8 for each point) for males and females, modified from [1]. Individual ulna length (from radiographs, 3×/week) and body mass (daily) were measured and growth was calculated through a least-squares linear regression over 20 days following the introduction of standard protein diet to the rehabilitation groups and age-matched animals from chronically fed standard (CT) and reduced protein (LP) groups. Rehabilitation intervals encompass the following durations: 1 = animals aged 40–49 days, 2 = 60–69 days, and 3 = 90–99 days (all comparisons *p* ≤ 0.001, except between LP and rehabilitated males). Results of *post hoc* comparisons of average growth between rehabilitated groups and CT/LP animals (ANOVA, with Bonferroni correction) and are indicated on the figure; CT comparisons above, LP below (*designates significance). Although ulna is provided as an example, tibia and humerus were also analyzed with very similar results. Of the three bones and body mass, only LP40 and CT male tibia growth for rehabilitation interval 1 were statistically indistinguishable, indicating catch-up growth followed the increased level of protein.

The second analysis examined isolated consumption pre- and post-rehabilitation times. This design is based on the analysis of Jones *et al* [1] where the bone sizes and body mass immediate pre and immediate post dietary rehab were compared (Figure 1). Here, we averaged each individual’s consumption within a 10 day window immediately pre- and post-rehabilitation, and tested for differences utilizing a mixed linear regression model, with *post hoc* tests conducted in the case of significance, utilizing a Bonferroni correction (as above). The first used the paired consumption rates of individuals to determine if pre- and post-rehabilitation consumption rates were different across the dietary modification and among treatment groups. The second compared (a) the three rehabilitated groups to LP animals, prior to rehab time and (b) the three rehabilitated groups to CT animals following the rehabilitation time. This tested (a) whether the LP control was a true control, as there should be no difference among the groups as they were on the same diet at that point, and (b) whether post-rehabilitation consumption matched control consumption.

## 3. Results and Discussion

### 3.1. Consumption through Experimental Period

**Figure 2:**
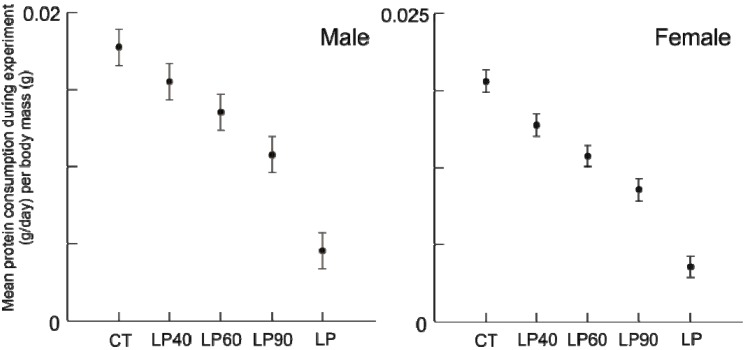
Average standardized protein consumption (g/day/body mass(g)) for males and females for the duration of the experiment (±standard deviation).

**Figure 3:**
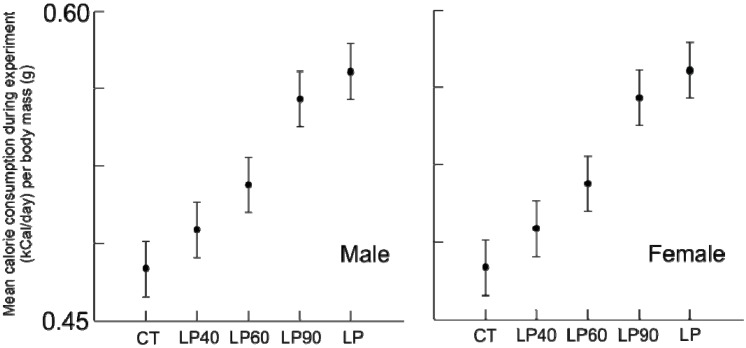
Average standardized calorie consumption (kCal/day/body mass (g)) for males and females for the duration of the experiment (±standard deviation).

The amount of protein (Figure 2) and calories (Figure 3) consumed during the experiment differed among treatment groups, for both sexes (*p* < 0.001). As expected, *post hoc* hypothesis testing confirmed that the longer animals were offered the LP diet, the amount of protein consumed was less (all comparisons, *p* ≤ 0.001), despite having access to additional food. Unlike protein consumption, calorie consumption was reduced. Despite being provided an isocaloric diet, animals fed reduced protein levels consumed greater calories than animals on the standard protein diet. Rehabilitation groups demonstrated a dose effect: animals with a shorter duration of protein malnutrition consumed fewer calories throughout the experiment than those malnourished longer (*post hoc* testing, *p* ≤ 0.001), with two exceptions: female LP90’s consumed nearly equal calories to chronic LP’s over the course of the experiment (*p* = 0.479) and female LP40’s were statistically indistinct from chronic CT’s (*p* = 0.054).

### 3.2. Consumption at Time of Rehabilitation (10 Day Averages)

**Figure 4:**
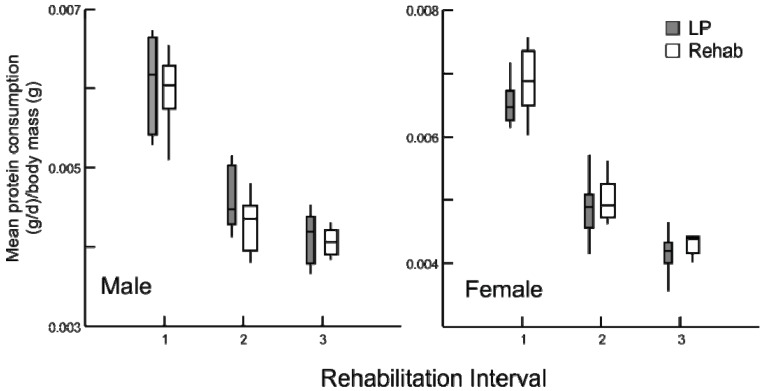
Daily average protein consumption (g/day) by body mass (g) for chronic LP and rehabilitation animals for 10 days prior to diet alteration. Rehabilitation intervals encompass the following durations: 1 = animals aged 30-39 days, 2 = 50–59 days, and 3 = 80–89 days (box plots with interquartile ranges indicated, *p* ≥ 0.5 for all comparisons).

Following the change in diet, all rehabilitation animals altered the amount of protein and calories consumed. The individual paired consumption rates of protein were different across the dietary modification and among treatment groups (*p* < 0.001). Prior to the switch in diet, all rehabilitated groups consumed the same amount of protein (Figure 4) and calories (data not presented) per body mass as the chronic LP animals, demonstrating their efficacy as a malnutrition control.

**Figure 5:**
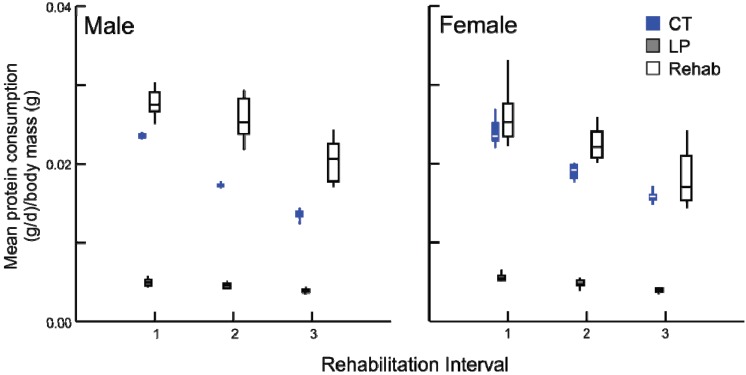
Box plots illustrating daily average protein consumption (g/day) by body mass (g) for chronic CT, LP and rehabilitation animals for 10 days following diet alteration. Rehabilitation intervals encompass the following durations: 1 = animals aged 40–49 days, 2 = 60–69 days, and 3 = 90–99 days (all comparisons *p* ≤ 0.001, except females at interval 1, where *p* = 0.685).

**Figure 6:**
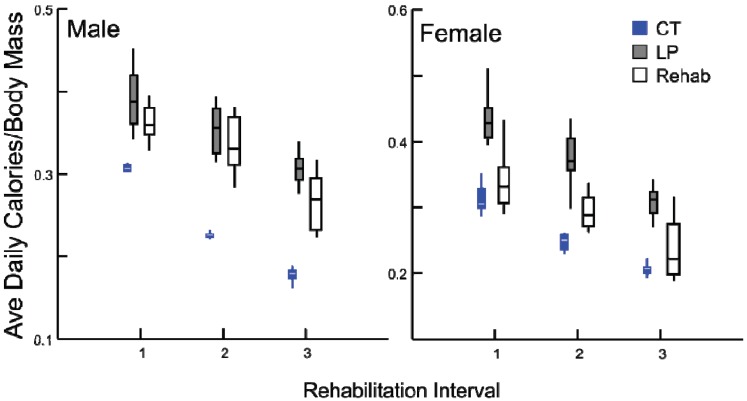
Box plots, illustrating daily average calorie consumption (kCal/day) by body mass (g) for chronic CT, LP and rehabilitation animals for 10 days following diet alteration. Rehabilitation intervals encompass the following durations: 1 = animals aged 40–49 days, 2 = 60–69 days, and 3 = 90–99 days (all comparisons *p* ≤ 0.001, except between LP and rehabilitated males).

After the dietary transition, the three rehabilitated groups consumed more protein than all LP animals and in some cases CT animals as well (Figure 5). Specifically, rehabilitated males consumed more protein than age-matched CT individuals (all tests *p* ≤ 0.001), but rehabilitated females consumed an equal amount of protein per body mass than did age-matched CT females (*p* = 0.685). The response of calorie consumption was varied depending upon age and sex (Figure 6). Regardless of age or sex, LP animals consumed more calories than CT animals (all comparisons *p* ≤ 0.001). At rehabilitation males’ caloric intake was indistinguishable from LP amounts, but females immediately decreased their caloric consumption, and the magnitude of change decreases with age.

### 3.3. Discussion

Despite some individual variation, there were no differences in consumption among the LP and rehabilitation groups prior to the change in diet (Figure 4). Therefore, differences in consumption immediately following the alteration of diet among the rehabilitation groups and LP animals provides an accurate assessment of pre- and post-rehabilitation consumption, even within a population of growing animals with daily, stochastic influences on satiation [6]. 

Consumption amounts change with age and body mass, and this was confirmed in both CT and LP groups. It is very important to consider this effect, and this is why we included age in our models. It is not possible to separate age and protein malnutrition duration with our model, but we did find age to be a significant factor in our results. All animals decreased calorie and protein consumption as they aged, including the rehabilitated groups. Also, despite increased calorie consumption in the LP animals, long bones length and body mass were often smaller than in CT or rehabilitated animals [1], although if given longer, those animals may have eventually attained larger sizes [2,3]. 

As protein was the manipulated factor, it was expected that protein consumption would change when the diet was altered. Although both males and females increased their protein consumption, it was interesting that males increased their consumption to greater than control levels, with a larger differential in the LP60 and LP90 groups. The significant interaction for male groups indicates that duration of protein malnutrition and/or age of animal matters more in males than in females. It is likely that this is the reason older rehabilitated males exceeded CT body mass by the end of the experiment [1]. These data do not, however, shed light on why the ulna would exceed CT lengths (in both males and females), where other long bones attained targeted sizes. What it does indicate is that some plasticity in bone growth and body mass exists and that different tissues are able to respond to nutritional alteration independently [3].

Protein content of the diet may influence how other nutrients are absorbed or processed. For instance, zinc and copper serum levels in children with protein energy malnutrition are lower than well-nourished children [7] and studies in humans have shown reduced calcium absorption with low protein diets [8] and our reduced protein diet contained a reduction in calcium carbonate because Dyets (Bethlehem, PA, USA) found problems associated with calcium depositions, particularly in the kidneys. However, protein complicates the absorption of polyphenols, as the amino acids show a biochemical affinity for these chemicals, making them less bioavailable [9]. It is clear that fluctuating protein in the diet will alter the processing of other nutrients, but with complex results.

Animals unexpectedly consumed fewer calories per unit body mass immediately after receiving the standard protein diet. This suggests that satiation is at least partially controlled by protein. Despite the decreased calorie consumption concordant with diet change, there was an associated increase in growth rate in all groups and sexes. As calories were decreased, this elevated growth rate must be fueled by the increase in protein consumption. This has important implications for the growing animal. Prior to the diet alteration, the rats experienced what is known as kwashiorkor-style malnutrition in humans [10]. This has different effects than calorie restriction, including disruption of the development of the nervous system, muscle wasting (including cardiac anomalies), atrophied intestinal mucosa, metabolic acidosis, fatty livers, and osteoporosis [10,11,12], in addition to mild general stunting [1,2,3,4,10]. The requirement for rehabilitative nutrition in this case focuses more on an increase in available amino acids, not an increase in calories. This has implications for interventions: rehabilitative diets should ameliorate the specific nutritional challenge and target the specific macronutrients needed to best support catch-up growth [10].

This study has several limitations. First, rats do not have strict determinant growth as humans do and their protein requirements are higher than humans. We conducted no histology or metabolic analyses through the rehabilitation time to assess what was functionally altered in order to accommodate increased growth while the animal was self-selecting fewer calories.

The literature on catch-up and targeted growth is confusing. Differences seen are dependent on length of insult, timing of insult, magnitude of insult, type of insult, species insulted. Many claim that the confusion has to do with various experimental procedures and a lack of continuity of methods, data capture, and measured parameters. Although true, it could also be that the mechanisms generating catch-up and targeted growth are multivariate and influenced by many, potentially cumulative and/or confounding factors that our current model is not able to discern. 

## 4. Conclusions

We measured total and protein consumption, body mass, and long bone length, following an increase of dietary protein at 40, 60 and 90 days, with two control groups (chronic LP or CT) for 150+ days. Immediately following rehabilitation, rats’ food consumption decreased significantly, implying that elevated protein intake is sufficient to fuel catch-up growth rates that eventually result in body weights and long bone lengths greater or equal to final measures of CT animals. The duration of protein restriction affected consumption: rats rehabilitated at younger ages had more drastic alterations in consumption of both calories and protein. While rehabilitated animals did compensate with greater protein consumption, variable responses in different ages and sex highlight the plasticity of growth and how nutrition affects body form.
